# Observations on Endo-Cardial Coagula

**Published:** 1850-07

**Authors:** Marcus Dana

**Affiliations:** Seneca County, Ohio


*Observations on Endo-Cardial Coagula.* By Marcus Dana, M. D., of Seneca county, Ohio.

The heart-clot, as cause of sudden death, has been almost entirely overlooked, and is probably of much more frequent occurrence than many of the Medical profession suppose.

Formerly, in the course of autopsies of persons who died suddenly, if either ventricle of the heart was found occupied by a recent coagulum, it was, generally, supposed to have been formed after the death of the individual.

The white clot, which has been noticed from time to time, was considered as a polypoid concretion, and was supposed to originate in a morbid condition of the heart itself; though in some French reports which I have seen recently there are some intimations of its true character.

A monograph was offered at the Concours for the chair *Pathologie Interne* at the Faculty of Paris, when MM. Piorry, Broussais, Dubois, and Gibert were candidates. M. Piorry was elected. The subject was, "Changes of the Blood in Disease," and some observations were made which have a bearing upon the subject under consideration, showing, however, that it was quite imperfectly understood at the time when those reports were made.

The polypiform concretions of the heart admitted by Malpighi, (oper. omn. 1666, de polypo cordis) and by many later writers, especially the philosopher and skilful practitioner Fr. Hoffmann, (Med. ration. Systemat. tom. 4, p. 3, 1738, Cap. 1, De Palpitatione Cordis,) and by the celebrated Vanswieten, (Comment. in Aphor. tom. 3, in 4to. 1758, §1010,) who ascribed to them the symptoms of grave organic lesions of the heart, were disputed by Morgagni, Senac, Pasta. Epist. de Cordis polypo in dubium revocat. Bergame 1739, and Lieutaud. Corvisart and Laennec hardly admitted their formation *during life*, except in some rare cases, and a *short time before death.* Recent facts have demonstrated that such concretions may exist during life.

M. Ferris at one of the sittings of the Academy of Medicine, (1828) reported that in a venesection, he had drawn from the vein of an arm, which became suddenly swollen and of a livid or blue color, a coagulum of considerable length. M. Velpeau exhibited at the same time to the same audience a vena cava filled by a central non-adherent coagulum, which had been arrested by the emulgent veins, and exhibited in some places a degeneration like medullary matter.

Bouillaud (Traité des Maladies du cœur) offers among other remarkable examples the curious fact of a heart, the right auricle of which was *nearly filled* with a *soft glutinous coagulum,* containing in its centre vesicles filled with a semi-concrete fluid, traversed by an infinite number of bright and dark red vessels.

M. Blaud de Beaucaire in a memoir in the 4th vol. 1833 of the "Révue Médicale," has collected a great number of facts derived from various authors and from his own practice, which go to establish the existence of these polypiform concretions of the heart. According to him many cases of *sudden* dyspnœa, deranged circulation, asthmatic affections, and palpitation of the heart, *appearing suddenly* and inexplicably, depend on such concretions *during life.*

The principal causes to which modern writers have attributed the formation of these clots, are the stasis of the blood during syncope, or organic lesions of the heart, or inflammation of its membranes. Among the late writers on this subject, (who are few) Prof. Meigs has contributed largely, and I believe is the first to call the attention of the profession in the United States to this subject, and I am not aware that any author on either side of the Atlantic has so clearly pointed out its true pathological character. He has established the fact that this clot can form in a moment during syncope under certain conditions of the system arising from great loss of blood, and that such an accident may cause death instantaneously if the clot is large, filling the pulmonic side of the heart. Or if partial, the action of the heart upon recovery from the syncope may press out the red corpuscles after a time, leaving the fibrinous portion retained within the cavity of the heart, constituting the *white clot.* In this case the individual may linger in a miserable existence for days, months, or years. On this particular point, he has given his views on sundry occasions before his Class in Jefferson Medical College, (which views he has entertained for many years) and also in an article which appeared in the March number of the "Medical Examiner," for 1849. To that interesting article the reader is referred particularly for the post mortem appearances there reported, as they go to establish the position he has taken.

This throws a flood of light upon the cause of many of those sudden deaths which every practitioner of experience must have observed to take place during or soon after syncope, from great loss of blood, particularly connected with parturition.

It is now admitted to be a fact, that the last portions of blood effused in an animal slowly bleeding to death, will coagulate much sooner than the first portions extravasated. The tendency to coagulation WITHIN the vessels, it will be inferred, will then be in a ratio to the loss of this vital fluid. What gives it this tendency in proportion to its exhaustion is not exactly known. The coagulation of the last portions mentioned, will take place much more quickly than the first, but the clot will be less firm. The coagulability at the natural temperature of the blood is due to the fibrin it contains. But the question that here presents itself is, what produces the TENDENCY to such a sudden coagulation? Carpenter says that "simple arrestment of nervous influence favors the coagulation of blood within the vessels; clots being formed a few minutes after the brain and spinal marrow have been broken down."

Is it not probable that the temporary paralysis of the small branches of the pneumogastric and sympathetic nerves (which are distributed to the inter-cellular passages and air cells of the lungs during syncope,) may have the principal agency in this phenomenon?" Thus the flow of blood through the lungs being arrested, the pulmonary artery is immediately distended with blood, and can receive no more from the heart. The vena cava continues pouring blood into the right auricle, and at this moment, by slowly distending it, the blood is converted into a clot.

"If a clot be formed in the right auricle, it will also be found in the iter ad ventriculum dextrum, filling up the cone of the tricuspid valve, and the nucleus of it will cause the coagulum to extend into the cavity of the right ventricle and pass to a greater or less distance along the tractus of the pulmonary artery. If the whole pulmonic side of the heart is occupied in this way, the death of the individual would be instantaneous."—(*Prof. Meigs.*) It will be seen that the first link in this chain of morbid phenomena is encephalic anæmia. It therefore follows that if this paralysis, dependent on anæmia of the encephalon, should in most cases be temporary, reaction would take place upon the application of proper means to restore the circulation to the brain, were it not for the mechanical interference with the heart's action; permanent asphyxia taking place, which might under other circumstances have been of a temporary character.

That coagulation does take place under these circumstances, is sufficiently proved by the white clot which is seen on examination of persons who have died under symptoms arising from a sudden, though not complete, interference with the due arterialization of blood, which in this, as well as other forms of asphyxia, is the immediate or proximate cause of death. And yet a sufficient amount of oxygen has in many such cases been furnished to support the system for an indefinite period of time, depending on the extent of coagulation.

Inferior animals undoubtedly would be found to perish in the same way, if it could be shown that great bodily exhaustion is productive of a condition of the nervous and vascular system similar to that developed in man. It is no uncommon circumstance to see that noble animal, the horse, (which a moment before, to all appearance, was in good health, excepting from exhaustion from severe muscular exercise,) fall and expire in a few moments, or partially recover and linger for some time, but never afterwards to be fit for service. He is said then to be "overheated" or "broken-winded." The case of my own horse, given hereafter, is supposed to be appropriate to the present question. It may go for what it is worth, to show that great bodily fatigue may produce the clot in question.

I will first proceed to describe a few cases of heart clot, as I am at present convinced they were, though at the time of occurrence of the first two mentioned, the cause was veiled in mystery, so far as my own knowledge went.

In the Fall of 1838 I was called to attend Mr. H., about 30 years of age, of good constitution, but who had been attacked some two or three weeks previously with nasal hæmorrhage, which recurring daily, had reduced him very much, so that he was compelled from weakness to keep the recumbent position. The hæmorrhage was produced by lifting heavy weights, producing determination to the head. I encountered much difficulty in arresting the epistaxis, but in a few days it ceased. He continued some days in a very debilitated state, and at length began slowly to convalesce. During this time his pulse was about 60 to 65 and regular, with respirations at 16—appetite good, bowels regular. I could discover no signs of organic or functional disorder about him.

In about a week from the cessation of the hæmorrhage he rose up for the first time, in the act of taking nourishment. Deliquium ensued immediately. He was replaced in the horizontal position, and partially recovered. At this time I was sent for, as he continued in great distress. I arrived in an hour and a half from the time of the fainting fit, but he had expired only a few moments before my arrival.

From his wife, a very intelligent woman, one who had had much experience as a nurse, I obtained the following history of the last symptoms of the man's case: After the fainting as above described, his respiration became laborious, with great heaving of the chest, and distress there. The pulse, which before he was raised was about 65, now became about 160, or, as she said, as fast as she could count. (She had been instructed to examine his pulse from time to time whilst the case was in my charge.) Coldness of the extremities; and great anxiety of countenance were noticed. These symptoms becoming more and more aggravated, he at length expired, as if by suffocation, accompanied by slight convulsive struggles, retaining his mental faculties till a short time before death. There was no blueness about the face, or turgidity of the vessels of the neck.

In August, 1840, I was called to attend Mrs. P. in her second accouchment, accompanying which no untoward circumstance occurred, save rather profuse hemorrhage in consequence of a too adherent placenta. She lost a considerable quantity of blood before the cause could be obviated, and had a fit of syncope. Reaction soon took place, the womb contracting to its normal size after labor, and becoming sufficiently firm, and the patient cheerful. I now considered her safe. The night being well spent, and my own residence some eight miles distant, I concluded to tarry till morning, and retired, charging my patient to keep herself perfectly still, as possibly a renewal of flooding might ensue. About an hour after this she insisted on getting up, in obedience to calls produced by some kind of purgative which she had taken in the early period of labor.

The ladies present tried in vain to persuade her to abide by my injunctions, and endeavor to manage without rising. She replied that "the Doctor is asleep and will not find it out," and succeeded in getting out of bed. Syncope took place immediately. The "Doctor" was aroused from his repose to witness one of the most appalling spectacles that can be imagined—the sudden and unexpected death of his patient. There were a few struggles, apparently to get breath; small vibrating or fluttering pulse, and cold extremities. She lived but a few minutes. In this case there had been but little hemorrhage from the time of the complete extraction of the placenta, and the womb was contracted to the normal size.

I then became convinced that there was great danger in suddenly changing the position of persons who were reduced by hæmorrhage, but what that danger really consisted in, was not easily defined. I NOW believe that this poor woman might have been saved by a simple anodyne injection, given immediately after delivery, to obviate the necessity of rising so soon.

Not having permission to examine the bodies of these persons, in consequence of superstitious prepossessions too prevalent in the country against disturbing the dead for the good of the living, I could only say, that they were cases of fatal syncope at the time of their occurrence.

In June, 1848, I attended Mrs. C. in her sixth confinement; she was a woman of good constitution, about 32 years of age, and had a good easy labor, but very profuse hemorrhage came on without any apparent cause, which continued rather profuse for several days. She became very much reduced. At length the hemorrhage ceased, and I supposed she was out of danger, if managed with discretion; and being under necessity of leaving home for three days, insisted that she should not be raised at all in my absence, and emphatically observed that there would be imminent danger in so doing. But in my absence her after pains became a little troublesome. A neighboring physician was called in who deemed it proper to administer cathartic medicine, during the operation of which she was forced on the close stool; syncope soon followed, from which she partially rallied. This took place a short time before my arrival at home, and I was immediately solicited to take charge again of the patient. Upon entering the room about two hours after the syncope, I found her laboring under the greatest difficulty of respiration. Every inspiratory action was produced by voluntary power, and very irregularly. I approached her and found a pulse scarcely perceptible, and numbering 150 in a minute, with cold extremities. I warned her husband of what I believed to be the approaching fate of his wife, and did not hesitate to declare that forcing her up had been the procuring cause of her forlorn condition; that a clot had formed at the instant of syncope, produced by suddenly changing her position, and would necessarily prove fatal.* Laudanum and acetate of lead injections were used to quiet the bowels, sinapisms, and warmth applied to the extremities, stimulants internally, galvanism—but all to no purpose, and I thought the stimulants internally given did harm.

The poor woman lingered along under these symptoms, the most horrible that can be imagined; the countenance expressive of the greatest anxiety and distress; saying, as long as the powers of speech remained, and after that by gesture, "Oh Doctor! do help me if you can." In short, she was anæmic from hæmorrhage. She took the cathartic in the morning, it forced her up at noon, in consequence of which, some great change took place in the respi-

* For this opinion I am indebted to the article before alluded to in the "Examiner."

ratory function, and at sun-set shé was where pain and trouble could not reach her.

I ought to have mentioned, that this woman told me herself, that she was very comfortable before attempting to get up, (save from after pains and physic.)

That great muscular efforts and consequent exhaustion of nervous power may induce those conditions which favor the sudden formation of clots in the heart, the following case of an animal would seem to prove:

One evening, in the autumn of 1848, while urging a horse of mine forward in great haste to see a person said to be alarmingly ill, and the animal having been used to excess during the day, he stopped short on the highway, staggered and came near falling, acting precisely like a person in a state of syncope; great difficulty of breathing came on at once; the poor fellow seemed to be in the utmost distress. He partially rallied, and I succeeded in leading him home, a distance of two or three miles at a very slow walk; for the instant I attempted to hurry him, symptoms of suffocation would ensue. His legs became cold at once; his tongue and mouth pale and bloodless; respiration laborious and hurried; very quick pulse. Thus he lingered on for several days in great distress, and became dropsical in the limbs before death.

Suspecting some affection of the heart or lungs, I proceeded to examine him soon after death. Upon opening his chest, the pericardium was found very much distended with serum. The left auricle and ventricle were empty, but the right ventricle was nearly filled with an enormous mass of white fibrinous matter, extending some portions into the right auricle. I could detect no other sign of disease in this animal. I had owned him two years, and he always carried the appearance of health until this sudden seizure.

I now leave this subject in the hands of the candid enquirer to form his own conclusions, and say if anything but the clot in question could account for the sudden deaths here related. Admitting this position, and the management of persons in a state of exhaustion from hæmorrhage or any other cause, will readily suggest itself to any intelligent person. Too much emphasis cannot be placed upon the propriety of confining such cases to the horizontal position until a sufficient amount of blood has been acquired to obviate the danger of syncope, and this can, in general, be determined by the force of the pulse, together with the general appearance of the patient. But even then their position should be gradually changed, examining closely the effects on the circulation.

